# Exoproteome Analysis of Antagonistic Interactions between the Probiotic Bacteria *Limosilactobacillus reuteri* LR1 and *Lacticaseibacillus rhamnosus* F and Multidrug Resistant Strain of *Klebsiella pneumonia*

**DOI:** 10.3390/ijms222010999

**Published:** 2021-10-12

**Authors:** Olga S. Savinova, Olga A. Glazunova, Konstantin V. Moiseenko, Anna V. Begunova, Irina V. Rozhkova, Tatyana V. Fedorova

**Affiliations:** 1 Research Center of Biotechnology, A. N. Bach Institute of Biochemistry, Russian Academy of Sciences, 119071 Moscow, Russia; savinova_os@rambler.ru (O.S.S.); olga.a.glas@gmail.com (O.A.G.); 2 Federal State Budgetary Scientific Institution “All-Russian Research Institute of Dairy Industry”, 115093 Moscow, Russia; abegunova@yandex.ru (A.V.B.); irina.v.rozhkova@gmail.com (I.V.R.)

**Keywords:** Lactobacilli, *Limosilactobacillus reuteri* LR1, *Lacticaseibacillus rhamnosus* F, *Klebsiella pneumonia*, multiple drug resistance, antagonistic interactions, co-cultivation, exoproteome

## Abstract

The expansion of multiple drug resistant (MDR) strains of *Klebsiella pneumoniae* presents an immense threat for public health. Annually, this microorganism causes thousands of lethal nosocomial infections worldwide. Currently, it has been shown that certain strains of lactic acid bacteria (LAB) can efficiently inhibit growth of *K. pneumoniae* and the formation of its biofilms; however, the active principle of such action remains unknown. In the current article, the growth inhibition of MDR *K. pneumoniae* by two LAB—*Limosilactobacillus reuteri* LR1 and *Lacticaseibacillus rhamnosus* F—is demonstrated, and the nature of this inhibition studied at the level of exoproteome. This article shows that the exoproteomes of studied LAB contains both classically and non-classically secreted proteins. While for *L. reuteri* LR1 the substantial portion of classically secreted proteins was presented by cell-wall-degrading enzymes, for *L. rhamnosus* F only one out of four classically secreted proteins was presented by cell-wall hydrolase. Non-classically secreted proteins of both LAB were primarily metabolic enzymes, for some of which a possible moonlighting functioning was proposed. These results contribute to knowledge regarding antagonistic interaction between LAB and pathogenic and opportunistic microorganisms and set new perspectives for the use of LAB to control the spread of these microorganisms.

## 1. Introduction

Currently, the large amount of antibiotics used in human medicine as well as in animal farming has resulted in the emergence and uncontrollable expansion of many multiple drug resistant (MDR) strains of pathogenic and opportunistic microorganisms [1]. Causing hospital-acquired (i.e., nosocomial) infections, these microorganisms are responsible for thousands of deaths worldwide [2,3,4]. This situation encourages scientists to search for antimicrobial therapeutic solutions as an alternative to antibiotics. In this respect, the use of beneficial microorganisms (i.e., probiotics) and their products (i.e., postbiotics) to eliminate potential pathogens and restore microbial balance is a recently established and highly promising field of research [5,6,7].

For a long time, lactic acid bacteria (LAB) were primarily studied with respect to food fermentation—the biochemical process that extends shelf life and increases the safety of products (e.g., milk, meat, fish and vegetables) while preserving and/or improving their main nutritional value [8,9,10]. It was originally thought that the main reason behind prolonged shelf-life and increased safety of fermented products is the presence of organic acids (i.e., lactic and acetic acid), alcohol and hydrogen peroxide produced by the fermenting LAB. In recent decades, it has become evident that LAB possess a more complex antagonistic system that inhibits growth of many pathogenic and opportunistic bacteria and fungi [11]. Although such aspects of this system as the production of bacteriocins, antimicrobial peptides and quorum sensing inhibitors have been demonstrated, many other aspects still remain to be explored [12,13,14].

*Klebsiella pneumoniae* is the infamous Gram-negative opportunistic pathogen. Causing severe nosocomial infections, *K. pneumoniae* is broadly recognized as a significant threat to global public health [15]. Being the paradigm of MDR, this bacterium not only displays high rate of acquisition of antibiotic resistance genes but also plays a pivotal role as a disseminator of these genes to other pathogenic and opportunistic microorganisms [16,17]. Moreover, the ability of *K. pneumoniae* to form bacterial biofilms embedded in a protective extracellular polymer matrix makes it highly resistant to patients’ immune defense mechanisms by skewing the immune response [18].

Currently, the inhibitory action of LAB on the growth of *K. pneumoniae* and the formation of its biofilms has been demonstrated by numerous studies [19,20,21,22]; however, the active principle of such action remains unknown. Moreover, the majority of research has been performed using cell-free supernatant (CFS) obtained from monocultures of different LAB. Such experimental design inevitably excludes antimicrobial substances produced by LAB upon induction by the presence of *K. pneumoniae*.

In this article, the growth inhibition of the MDR *K. pneumoniae* strain during its two- and three-species co-cultivation with *Limosilactobacillus* reuteri LR1 and *Lacticaseibacillus rhamnosus* F is demonstrated, and the nature of this inhibition studied at the protein level. Following this, a mass spectrometric (MS) analysis of exoproteomes from monocultures of *L. reuteri* LR1 and *L. rhamnosus* F as well as from all their co-cultivations with *K. pneumoniae* is performed. To identify proteins from the obtained MS data, the genomes of *L. reuteri* LR1 and *L. rhamnosus* F sequenced and annotated in this study were used.

## 2. Results

### 2.1. Sequencing and Annotation of L. reuteri LR1 and L. rhamnosus F Genomes

Using Ion Torrent technology, the draft genomes of *L. reuteri* LR1 and *L. rhamnosus* F were sequenced with overall coverage of 100× and ultimately assembled into 319 and 57 contigs, respectively (Table 1). For *L. reuteri* LR1, the N50 value was 20,552 bp with the longest contig being 64,232 bp and the mean contig size 6437 bp. For *L. rhamnosus* F, the N50 value was 144,365 bp with the longest contig being 310,149 bp and the mean contig size 44,023 bp. The final size of the assemblies comprised 2.1 and 2.9 Mb for *L. reuteri* LR1 and *L. rhamnosus* F, respectively. The Whole Genome Shotgun projects were deposited at DDBJ/ENA/GenBank under the accessions JAHLXI000000000 and JAHLXH000000000 for *L. reuteri* LR1 and *L. rhamnosus* F, respectively. The versions described in this paper are versions JAHLXI000000000.1 and JAHLXH000000000.1 for *L. reuteri* LR1 and *L. rhamnosus* F, respectively.

In the assembled genome of *L. reuteri* LR1, a total of 2179 genes were predicted, of which 1947 were identified as protein-coding, 83 as RNA-coding, and 149 as pseudogenes. In the assembled genome of *L. rhamnosus* F, a total of 2736 genes were predicted, of which 2607 were identified as protein-coding, 71 as RNA-coding, and 85 as pseudogenes. There were no CRISPR arrays detected in the *L. reuteri* LR1 genome, while the genome of *L. rhamnosus* F contained one CRISPR array.

**Figure 1:**
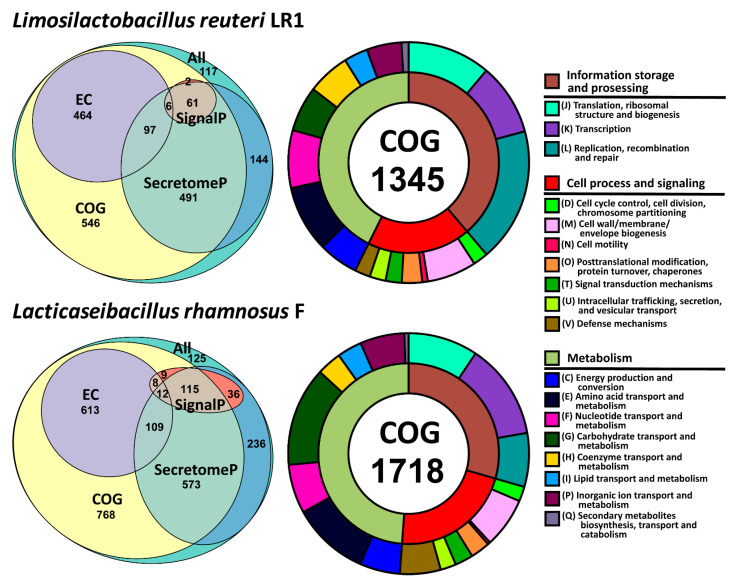
Functional annotation of the *L. reuteri* LR1 and *L. rhamnosus* F genomes. General results of the eggNOG, SignalP and SecretomeP annotations are summarized on the area-proportional Euler diagrams (please, note that some intersections can be excluded to achieve the trade-off between accuracy of the intersection areas and meaningful arrangement of the ellipses). Information about the clusters of orthologous groups (COG) content of the genomes, excluding nonspecific COG categories (i.e., R, S and X), are summarized on the double-layer donut charts.

Functional annotation of the predicted protein-coding genes was performed using the orthologous groups database eggNOG [23], and the prediction of signal peptides and possible secretion was performed with SignalP [24] and SecretomeP [25], respectively (Figure 1, Supplementary Tables S1 and S2). As a result of general functional prediction, 1669 genes (86%) of *L. reuteri* LR1 and 2207 genes (85%) of *L. rhamnosus* F received clusters of orthologous groups (COG) functional categories—1345 (69%) and 1718 (66%) upon exclusion of nonspecific COG categories (i.e., R, S and X), respectively. For *L. reuteri* LR1, 570 genes (29%) were assigned to a specific Enzyme Commission number (EC number), and for *L. rhamnosus* F this number was received by 743 genes (28%). In the *L. reuteri* LR1 genome, 88 proteins (4%) containing signal peptides were determined, and for 732 proteins (38%) possible non-classical secretion was predicted (these proteins do not contain signal peptides but can be secreted according to SecretomeP). In the *L. rhamnosus* F genome, 182 proteins (7%) containing signal peptides were determined, and for 919 (35%) proteins possible non-classical secretion was predicted.

### 2.2. Co-Cultivation of L. reuteriLR1 and L. rhamnosus F with Multidrug Resistant K. pneumonia

**Figure 2:**
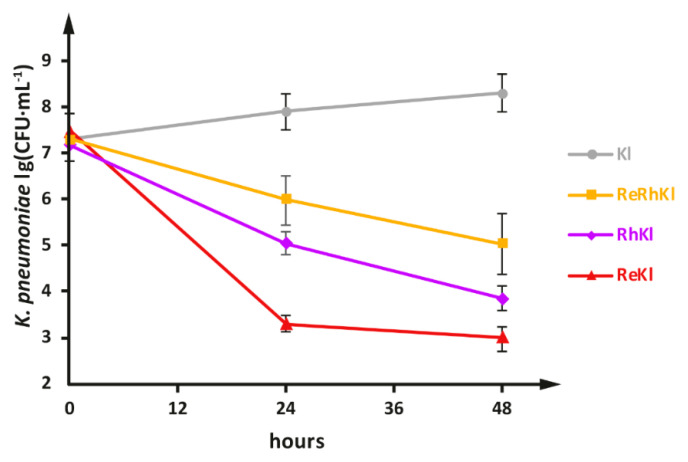
Changes in the viable cell count of *K. pneumoniae* during single-, two- and three-species cultivations with *L. reuteri* LR1 and *L. rhamnosus* F. Cultivations: **Kl**, *K. pneumoniae* monoculture; **ReRhKl**, *L. reuteri* LR1, *L. rhamnosus* F and *K. pneumoniae* three-species co-cultivation; **RhKl**, *L. rhamnosus* F and *K. pneumoniae* two-species co-cultivation; and **ReKl**, *L. reuteri* LR1 and *K. pneumoniae* two-species co-cultivation.

To examine antagonistic activity of *L. reuteri* LR1 and *L. rhamnosus* F against planktonic cells of the MDR clinical isolate of *K. pneumoniae*, two two-species co-cultivations, *L. reuteri* LR1 with *K. pneumoniae* (**ReKl**), and *L. rhamnosus* F with *K. pneumoniae* (**RhKl**), and one three-species co-cultivation, *L. reuteri* LR1 and *L. rhamnosus* F with *K. pneumoniae* (**ReRhKl**), were performed. The single-species cultivation of *K. pneumoniae* was used as a control. The dynamics of changes in the viable cell count are shown in the Figure 2.

While slow growth of *K. pneumoniae* was observed in its monoculture, in co-cultivations with LAB the viable cell count of *K. pneumoniae* constantly decreased. The most prominent decrease was observed in the case of **ReKl** co-cultivation, during which the viable cell count of *K. pneumoniae* steeply decreased by approximately 4 orders of magnitude after 24 h of co-cultivation and remained at the same level up to 48 h of cultivation. In the **RhKl** co-cultivation, viable cell count of *K. pneumoniae* declined less sharply, by 2 and 3 orders of magnitude after 24 and 48 h of co-cultivation, respectively. Surprisingly, during **ReRhKl** co-cultivation the smallest decrease in viable cell count of *K. pneumoniae* was observed, as compared with the **ReKl** and **RhKl** co-cultivations, by 1 and 2 orders of magnitude after 24 and 48 h of cultivation, respectively.

**Figure 3:**
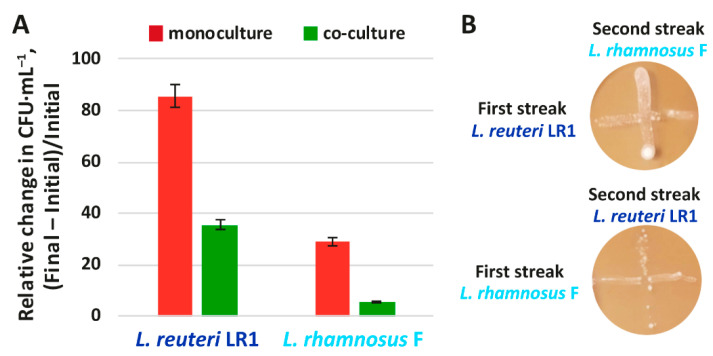
(**A**) Relative changes of *L. reuteri* LR1 and *L. rhamnosus* F viable cell count during single- and two-species cultivations; (**B**) Testing of interaction between *L. reuteri* LR1 and *L. rhamnosus* F by perpendicular streak method.

It may be hypothesized that the less prominent suppression of *K. pneumoniae* during **ReRhKl** co-cultivation was related to the antagonistic interactions between LAB. To substantiate this hypothesis, two additional experiments were performed. Firstly, the growth of *L. reuteri* LR1 and *L. rhamnosus* F during their liquid-state single-species cultivation was compared with that during the two-species co-cultivation of these LAB (**ReRh**) (Figure 3A). Secondly, the interaction between LAB during their solid-state co-cultivation was tested by the perpendicular streak method (Figure 3B).

Both liquid- and solid-state experiments demonstrated that although *L. reuteri* LR1 and *L. rhamnosus* F did not show pronounced antagonism, they inhibited the intensive growth of each other. While during single-species liquid-state cultivation relative changes of viable cell count for *L. reuteri* LR1 and *L. rhamnosus* F were 86 and 29 times in 24 h, during **ReRh** co-cultivation they were 35 and 6 times, respectively (Figure 3A). When tested on a solid agar medium, both LAB did not show strong zones of growth inhibition. However, near the zone of contact (intersection of two streaks), the least intensive growth was observed for both LAB (Figure 3B).

### 2.3. Compositional Analysis of Exoproteomes

In order to identify proteins differentially secreted by LABs, cell-free cultural liquid was collected from all mentioned cultivations (i.e., **Re**, **Rh**, **ReKl**, **RhKl**, **ReRh** and **ReRhKl**) at the 24 h time point. The samples were concentrated; proteins were precipitated, separated by 2DE and analyzed by MALDI TOF/TOF MS/MS. The analyzed proteins were matched with those annotated in the genomes of *L. reuteri* LR1 and *L. rhamnosus* F.

**Figure 4:**
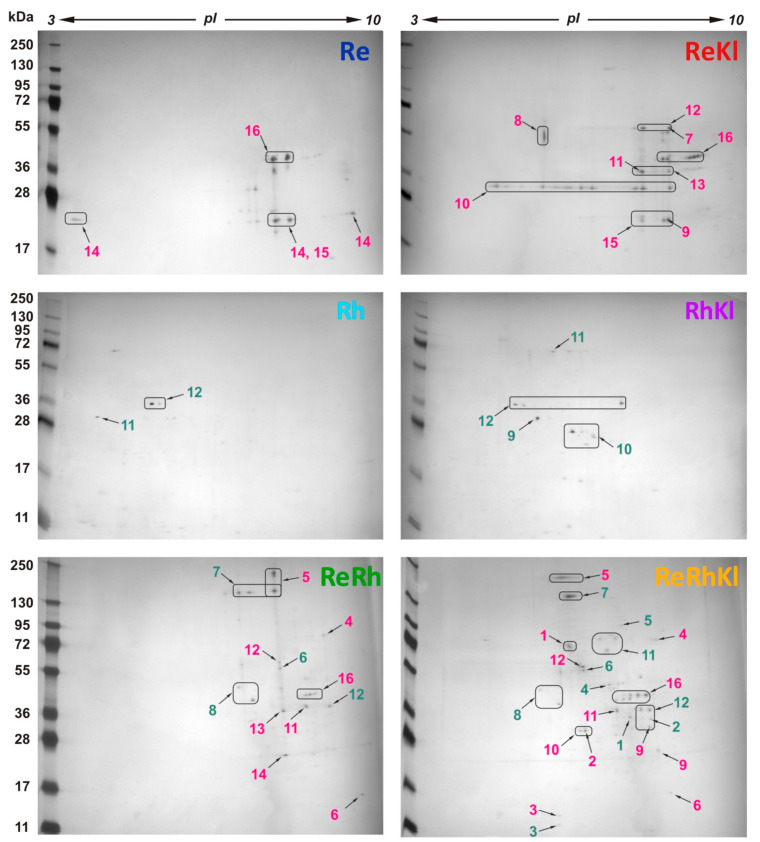
The results of two-dimensional gel electrophoresis (2-DE). The proteins belonging to *L. reuteri* LR1 are designated in red and to *L. rhamnosus* F in green. The result of protein identifications is presented in Figure 5. Cultivations: **Re**, *L. reuteri* LR1 monoculture; **ReKl**, *L. reuteri* LR1 and *K. pneumoniae* two-species co-cultivation; **Rh**, *L. rhamnosus* F monoculture; **RhKl**, *L. rhamnosus* F and *K. pneumoniae* two-species co-cultivation; **ReRh**, *L. reuteri* LR1 and *L. rhamnosus* F two-species co-cultivation; **ReRhKl**, *L. reuteri* LR1 and *L. rhamnosus* F and *K. pneumoniae* three-species co-cultivation.

**Figure 5:**
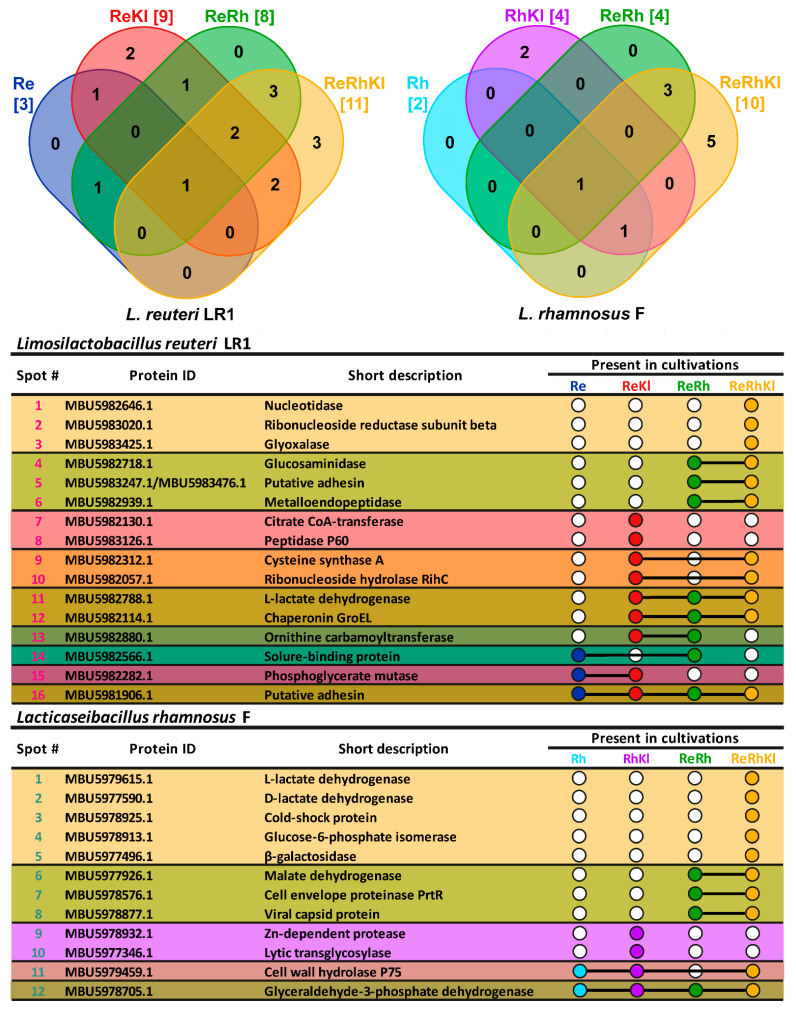
Proteins that were identified on 2-DE gels (Figure 4) are listed in the table. The collective presence of the proteins in the different cultivations is depicted on the Venn diagrams (**panel’s top**), while the individual presence is depicted as UpSet-style plot (**panel’s bottom**). On the UpSet-style plot, the presence of a protein in the corresponding cultivation is marked by a colored circle, if that protein was detected in several cultivations, the corresponding circles are joined by a solid line. Cultivations: **Re,** *L. reuteri* LR1 monoculture; **ReKl**, *L. reuteri* LR1 and *K. pneumoniae* two-species co-cultivation; **Rh**, *L. rhamnosus* F monoculture; **RhKl**, *L. rhamnosus* F and *K. pneumoniae* two-species co-cultivation; **ReRh**, *L. reuteri* LR1 and *L. rhamnosus* F two-species co-cultivation; **ReRhKl**, *L. reuteri* LR1 and *L. rhamnosus* F and *K. pneumoniae* three-species co-cultivation.

For all cultivations, analysis of 2D-gels revealed approximately 100 spots that were assigned by MALDI TOF/TOF MS/MS to 28 unique proteins, 16 proteins from the genome of *L. reuteri* LR1 and 12 proteins from the genome of *L. rhamnosus* F (Figure 4 and Figure 5). It should be noted that after manual curation two proteins of *L. reuteri* LR1, MBU5983247.1 and MBU5983476.1 (both are products of incomplete open reading frames), turned out to be parts of a single protein whose gene was misassembled due to the presence of a highly repetitive region. Hence, for further analysis this protein was abbreviated as MBU5983247.1/MBU5983476.1. Importantly, no proteins belonging to *K. pneumoniae* were detected in any cultivation. By their nature, proteins secreted by *L. reuteri* LR1 and *L. rhamnosus* F were extremely different. Only one type of protein, L-lactate dehydrogenase, was secreted by both LAB. For *L. reuteri* LR1 this protein was secreted during **ReKl**, **ReRh**, and **ReRhKl** co-cultivations, while for *L. rhamnosus* F it was secreted only during **ReRhKl** co-cultivation.

For both LAB the greatest number of secreted proteins was detected during **ReRhKl** co-cultivation, 11 for *L. reuteri* LR1 and 10 for *L. rhamnosus* F; while the lowest was detected during their growth in monoculture (i.e., **Re** and **Rh**), three for *L. reuteri* LR1 and two for *L. rhamnosus* F (Figure 4 and Figure 5). For two-species co-cultivations of *L. reuteri* LR1, nine proteins were detected during **ReRh** co-cultivation and eight during **ReKl** co-cultivation. For both two-species co-cultivations of *L. rhamnosus* F (i.e., **RhKl**, **ReRh**), four proteins were detected. In comparison with *L. rhamnosus* F, *L. reuteri* LR1 was characterized by the greater number of proteins present, which was detected in several co-cultivations (eleven vs. five). At the same time, *L. rhamnosus* F secreted a greater number of co-cultivation specific proteins (seven vs. five for *L. reuteri* LR1). Importantly, there were no monoculture (i.e., **Re** and **Rh**) specific proteins detected for both LAB, and all co-cultivation specific proteins were detected upon the presence of *K. pneumoniae* (i.e., **ReKl**, **RhKl** and **ReRhKl**).

### 2.4. Functional Analysis of Exoproteomes

For the proteins that were identified in all the exoproteomes, the NCBI and eggNOG functional annotations were joined and manually curated (Table 2). For both LAB approximately 50% of the identified proteins (ten for *L. reuteri* LR1 and six for *L. rhamnosus* F) were involved in different metabolic processes. All these proteins were metabolic enzymes, with the only exception being the solute-binding protein (MBU5982880.1) of *L. reuteri* LR1 that participates in the active transport of amino acids across the cytoplasmic membrane. Most of the determined metabolic enzymes (eight for *L. reuteri* LR1 and five for *L. rhamnosus* F) were primarily intracellular. The only extracellular metabolic enzymes were nucleotidase (MBU5982646.1) and β-galactosidase (MBU5977496.1) of *L. reuteri* LR1 and *L. rhamnosus* F, respectively. Additionally, possible non-classical secretion was determined for ribonucleoside reductase (MBU5983020.1) of *L. reuteri* LR1 and glyceraldehyde-3-phosphate dehydrogenase (MBU5978705.1) of *L. rhamnosus* F.

In the exoproteomes of *L. reuteri* LR1, the main part of the metabolic enzymes was involved either in the amino acid transport and metabolism (COG E, three proteins) or nucleotide transport and metabolism (COG F, three proteins). In comparison, the main part of the metabolic enzymes secreted by *L. rhamnosus* F was involved either in energy production and conversion (COG C, three proteins) or carbohydrate transport and metabolism (COG G, three proteins).

In addition to the metabolically active proteins, the exoproteomes of both LAB contained several cell wall-degrading enzymes (three for *L. reuteri* LR1 and two for *L. rhamnosus* F). All these enzymes contained signal peptides and, hence, were secreted via classical secretion pathways. The only exception was lytic transglycosylase (MBU5977346.1) of *L. rhamnosus* F for which possible non-classical secretion was determined.

In the exoproteomes of *L. reuteri* LR1, two functional groups of proteins absent in the exoproteomes of *L. rhamnosus* F were identified. The first group contained two proteins participating in cell wall adhesion (i.e., adhesins), and the second group were presented by Chaperonin GroEL that promotes the refolding and proper assembly of unfolded polypeptides. The unique for *L. rhamnosus* F functional groups of proteins were presented by two proteases and one cold-shock protein that participate in post-translational gene regulation. Importantly, in the exoproteomes of *L. rhamnosus* F one protein belonging to the viral capsid (MBU5978877.1) was identified.

## 3. Discussion

In summary, the performed two- and three-species co-cultivation of MDR *K. pneumoniae*, *L. reuteri* LR1 and *L. rhamnosus* F demonstrated the antagonistic effects among these bacteria. While *L. reuteri* LR1 and *L. rhamnosus* F were able to suppress the growth of *K. pneumoniae* by 2–4 orders of magnitude during their two-species co-cultivations (i.e., **ReKl** and **RhKl**), in the three-species co-cultivation (i.e., **ReRhKl**) the growth of *K. pneumoniae* was suppressed only by 1–2 orders of magnitude. The latter was explained by the observed weak antagonistic effect between *L. reuteri* LR1 and *L. rhamnosus* F. Hence, the obtained data suggest that, at least *in vitro*, the mixture of *L. reuteri* LR1 and *L. rhamnosus* F is inferior in terms of *K. pneumoniae* growth suppression to the single-strain probiotic culture; however, whether or not this tendency will be observed during *in vivo* administration of probiotics must be independently confirmed. Although there is a current tendency to mix as many probiotic strains as possible into a single product, our data strongly support the recent claim that the choice of an appropriate probiotic product should not be based on the number of strains in it but rather on evidence-based trials of the product’s efficacy in a given situation [26].

The obtained assembly and annotation of the draft genomes of *L. reuteri* LR1 and *L. rhamnosus* F were of comparable quality with those previously published [27]. Although each genome provides an enormous amount of information, in this article the obtained genomes were primarily used as the mean to unambiguously identify proteins present in the studied exoproteomes. Besides the fact that exoproteomes of LAB grown in the presence of *K. pneumoniae* were never previously reported, the main rationale for its investigation with respect to the antagonistic interactions between studied strains were as follows: (1) since both LAB and *K. pneumoniae* are acid-tolerant bacteria [28,29], all possible non-specific acid-related bactericidal effects play a negligibly small role during their interactions; (2) although production of bacteriocins can play a role in antagonistic interactions between studied LAB, the hypothesis of a bacteriocins-related inhibition of *K. pneumoniae* growth seems unlikely; it is generally accepted that Gram-negative bacteria possess a natural resistance to the bacteriocins synthesized by Gram-positive bacteria [30,31]; (3) there is an ever growing body of evidence that certain secreted proteins of LAB could be responsible, among others, for their pathogen-inhibitory properties [32,33,34].

In general, all proteins detected in the exoproteomes of both LAB can be classified into those that are primarily extracellular (i.e., can undergo classical secretory pathways due to the presence of signal peptides) and those that are primarily intracellular. It should be noted that in the current study cell lysis was not observed; hence, it may be proposed that the detection of primarily intracellular proteins in the exoproteomes is a result of their non-classical secretion, as was previously discussed in [35]. The obtained data show substantial limitations of SecretomeP software [25] for prediction of non-classically secreted proteins in LAB, since it failed to predict this secretion for many apparently non-classically secreted proteins (Table 2).

**Figure 6:**
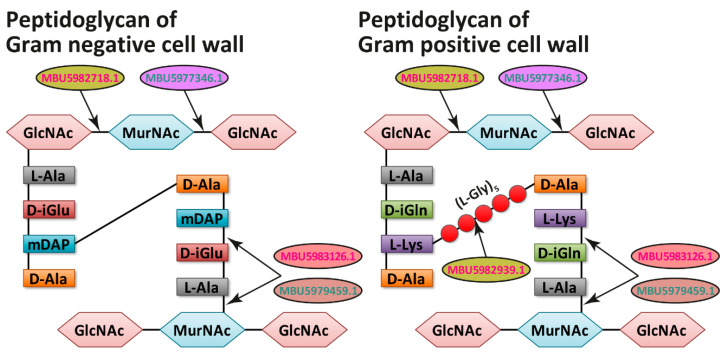
Cell wall-degrading enzymes of *L. reuteri* LR1 (red) and *L. rhamnosus* F (green) determined in this study (Figure 4 and Figure 5 and Table 2).

In the case of *L. reuteri* LR1, the substantial portion of classically secreted proteins (3 out of 7) was presented by cell wall-degrading enzymes (Table 2). Although these enzymes play a significant role in the growth and division of *L. reuteri* LR1, it may be proposed that in co-cultivations they can participate in antagonistic interactions by disrupting the cell walls of neighboring bacteria [36]. Upon the presence of *L. rhamnosus* F in co-cultivation (**ReRh** and **ReRhKl**), *L. reuteri* LR1 secreted such cell wall-degrading enzymes as *N*-acetylglucosaminidase (Glycosyl hydrolase family 73, GH73) (MBU5982718.1) and metalloendopeptidase (family M23) (MBU5982939.1) (Table 2). While *N*-acetylglucosaminidase (MBU5982718.1) hydrolyses the glycosidic bonds in the peptidoglycan (PG) of both Gram-positive and Gram-negative bacteria, peptidase M23 (MBU5982939.1) cuts the peptide bond of the PG cross-link in Gram-positive bacteria (Figure 6). Although, the involvement of peptidase of family M23 in antagonistic interaction between *Lactobacillus salivarius* and *Staphylococcus aureus* has been previously reported [37], this is the first report on the possibility of the involvement of these peptidases in the antagonistic interactions between different LAB species. Upon the presence of *K. pneumoniae* in co-cultivation (**RhKl**), *L. reuteri* LR1 secreted Peptidase P60 (MBU5983126.1) containing NlpC/P60 domain. Previously it has been demonstrated that NlpC/P60 domain-containing peptidases can catalyze the hydrolysis of both Gram-positive and Gram-negative bacterial PG [38] (Figure 6). Among the remaining classically secreted proteins of *L. reuteri* LR1, two proteins were putative adhesins (MBU5983247.1/MBU5983476.1 and MBU5981906.1), one a solute binding protein and the other a nucleosidase. While the secretion of MBU5983247.1/MBU5983476.1 was linked with the presence of *L. rhamnosus* F in co-cultivation (**ReRh** and **ReRhKl**), the secretion of MBU5981906.1 was observed on every cultivation (**Re**, **ReKl**, **ReRh** and **ReRhKl**). It may be hypothesized, that the secretion of these adhesins was stimulated by the expected competition over adhesion to the surfaces of possible host cells; hence, in the current experimental setup it can be regarded as an artifact that did not contribute to the observed antagonistic interactions. The presence of solute-binding protein in monoculture (**Re**) and co-cultivation with *L. rhamnosus* F (**ReRh**) can be explained by the active growth of *L. reuteri* LR1 in the former case and the competition for substrate in the latter. The presence of nucleosidase in three-species co-cultivation (**ReRhKl**) can also be explained by the competition for the substrate among microorganisms.

In the case of *L. rhamnosus* F, only one out of four classically secreted proteins was a cell wall-degrading enzyme, cell wall hydrolase P75 (MBU5979459.1), also known as major secreted protein 1 [39], containing NlpC/P60 domain (Figure 6). Although this hydrolase was present in monoculture (**Rh**), in co-cultivations its secretion can be associated with the presence of *K. pneumoniae* (**RhKl** and **ReRhKl**). Also, it is worth mentioning here the secretion, although non-classical, of lytic transglycosylase (MBU5977346.1) in the co-cultivation with *K. pneumoniae* (**RhKl**). This enzyme can catalyze the hydrolysis of both Gram-positive and Gram-negative bacterial PG (Figure 6). The presence of a detectable quantity of cell envelope proteinase PrtR only upon co-cultivation with *L. reuteri* LR1 (**ReRh**) and *β*-galactosidase only in the three-species co-cultivation (**ReRhKl**) may be explained by active competition between microorganisms for the substrates (amino acids and sugars, respectively) during these co-cultivations. As for the adhesins secreted by *L. reuteri* LR1, the secretion of Zn-dependent protease (peptidase family M10), which is homologous to the human matrix metallopeptidase and is capable of degrading extracellular matrix proteins, in the co-cultivation with *K. pneumoniae* (**RhKl**) was most probably stimulated by the expected competition for adhesion to the host cells.

Since the main part of the non-classically secreted proteins found in the exoproteomes of both LAB was primarily metabolic enzymes, it may be proposed that in the observed situation these proteins demonstrated a moonlighting functionality. By definition, moonlighting proteins are proteins that apart from their well-characterized function can perform other physiologically relevant biochemical or biophysical activities, frequently, in different cell compartments and at different times [40,41,42]. Typically, intracellular/extracellular moonlighting proteins are “housekeeping proteins” that normally participate in the central processes of carbohydrate, protein and nucleic acid metabolism [43]. As in the case of many other microorganisms, there is a limited amount of knowledge regarding the moonlighting proteins of LAB. The main part of known LAB moonlighting proteins has been previously associated with the host–probiotic interaction, and the most well-known examples are GAPDH and enolase [44,45,46].

As it was already mentioned with regard to the classically secreted adhesins of *L. reuteri* LR1 and Zn-dependent protease of *L. rhamnosus* F, although secretion of moonlighting proteins that promotes host-probiotic interaction was most probably induced by the presence of the neighboring microorganisms, in the current experimental setup this secretion can be regarded as an artifact since no host to interact with was present. Nevertheless, moonlighting activities different from the host-probiotic interaction can be proposed for several proteins based on the previously published data.

For *L. reuteri* LR1, the possible moonlighting function can be proposed for Cysteine synthase A (CysK) whose secretion can be associated with the presence of *K. pneumoniae* (**ReKl** and **ReRhKl**). CysK typically carries out the second step in the pathway of cysteine biosynthesis, synthesizing cysteine from sulfide (H_2_S) and *O*-acetyl-L-serine; however, it has been shown that in certain bacteria, including *Lactobacillus casei*, this enzyme can perform cysteine desulfurization leading to the formation of pyruvate, ammonia (NH_3_) and H_2_S [47]. Recent investigations demonstrate that the presence of both NH_3_ and H_2_S during cultivation significantly influenced bacterial physiology [48,49]. As an example, it has been previously shown that production of NH_3_ by *Proteus mirabilis* gives it a competitive advantage over *K. pneumoniae* [50].

For *L. rhamnosus* F, the possible moonlighting function may be proposed for d-lactate dehydrogenase. The d-lactate dehydrogenase (D-LDH/LdhD-1, YP_003169904.1) of *L. rhamnosus* GG, homologous to that (MBU5977590.1) detected in the exoproteome of *L. rhamnosus* F during three-species co-cultivation (**ReRhKl**), was previously recombinantly expressed in *Escherichia coli* [51]. This protein has a 91.3 % similarity with the D-lactate dehydrogenase (HicDH, AAA25236.1) of *Lactobacillus casei*, which plays the main role in the formation of D-lactic acid [52]. However, most probably in *L. rhamnosus* it plays a different role, since its activity by pyruvate is very low [51]. There is a high probability that this enzyme can perform a D-2-hydroxy acid dehydrogenase function producing phenyllactic acid from phenylpyruvate [53,54]. The phenyllactic acid is a natural biological antimicrobial agent that has been previously shown to effectively inactivate *Klebsiella oxytoca* planktonic and biofilm cells [55].

Among all non-classically secreted proteins of *L. rhamnosus* F, special attention should be devoted to the viral capsid protein (MBU5978877.1) secretion of which has been linked with the presence of *L. reuteri* LR1 in co-cultivation (**ReRh** and **ReRhKl**). Although in the genome of *L. rhamnosus* F this protein is part of an integrated prophage, there were no additional phage proteins of this prophage detected in any exoproteome. Hence, it can be concluded that the gene encoding MBU5978877.1 is a “moron locus” [56]. While the majority of phage-encoded genes are repressed during lysogeny, moron loci are often highly expressed and can provide a competitive advantage to the host [57]. Interestingly, the secretion of individual prophage genes was previously reported for *L. rhamnosus* LRB [58], while *L. rhamnosus* Pen can spontaneously release entire phage particles that were proposed to increase the survivability of microorganism in their natural ecological niche [59].

## 4. Materials and Methods

### 4.1. Bacterial Strains and Pre-Cultivations

Strains of *L. reuteri* LR1 and *L. rhamnosus* F were obtained from the Microorganism Collection of the All-Russia Research Institute of the Dairy Industry (VNIMI, Moscow, Russia). The sequences of the 16S ribosomal RNA genes of these strains can be found at the GeneBank accessory numbers MN994628 and MN994629 for *L. reuteri* LR1 and *L. rhamnosus* F, respectively.

The hospital strain isolate of *K. pneumoniae* was obtained from the V.I. Shumakov Federal Research Center of Transplantology and Artificial Organs (Moscow, Russia). The strain was isolated from an endotracheal tube and its antibiotic resistance profile was determined with MicroScan WalkAway 96 plus system (Beckman Coulter, Atlanta, GA, USA) using MicroScan Neg Breakpoint Combo Panel Type 44 (NBC44). The strain demonstrated resistance to 24 antibiotics in the panel including: ampicillin, ampicillin/sulbactam, amoxicillin/clavulanic acid, piperacillin, piperacillin/tazobactam, cefazolin, cefuroxime, cefoxitin, cefotaxime, ceftazidime, ceftriaxone, cefepime, ciprofloxacin, levofloxacin, meropenem, imipenem (tienam), ertapenem, amikacin, gentamicin, tobramicine, aztreonam, tetracycline, tigecycline (tygacil), trimetoprim/sulfometoxazol [60].

For inoculum preparation, LAB and hospital strains of *K. pneumoniae* were cultivated under anaerobic conditions on MRS (De Man, Rogosa and Sharpe) broth at 37 ± 1 °C to achieve the turbidity of 3 McFarland standard and stored at 4 °C until further use; the viable cell counts were measured (as described below in the Section 4.3 and Section 4.4) and adjusted to 10^8^ CFU·mL^−1^ by dilution with MRS broth before inoculations.

### 4.2. DNA Isolation, Sequencing and Annotation

For DNA extraction, *L. reuteri* LR1 and *L. rhamnosus* F were statically cultivated in 20 mL of MRS broth at 37 ± 1 °C until cloudy in appearance. The approximately 0.1 mL of bacterial cells were separated by centrifugation at 10,000× *g* for 5 min at 4 °C, pretreated with 20 µL of lysozyme solution (50 mg·mL^−1^) (Serva, Heidelberg, Germany) and 5 µL of RNase A (2 mg·mL^−1^) (Fermentas, St. Leon-Rot, Germany) at 37 °C for 20 min, and total DNA was extracted using DNeasy mericon Food Kit (Qiagen, Valencia, CA, USA), according to the manufacturer’s protocol. The quality and quantity of the isolated DNA were checked using an Agilent Bioanalyzer 2100 (Agilent Technologies, Foster City, CA, USA) and Qubit fluorimeter (Thermo Fisher Scientific, Waltham, MA, USA).

The DNA library was prepared using the Ion AmpliSeq library kit 2.0 (Thermo Fisher Scientific, MA, USA) and indexed with an Ion Xpress barcode adapters 1–16 kit (Thermo Fisher Scientific, MA, USA). The quality and quantity of the obtained DNA library were checked using Agilent Bioanalyzer 2100. Whole genome sequencing was carried out using the Ion Torrent Personal Genome Machine (PGM) (Thermo Fisher Scientific, MA, USA). The obtained reads were pre-processed and assembled with CLC Genomics Workbench 11.0 (Qiagen, Valencia, CA, USA). Upon submission, genome annotations were performed using NCBI Prokaryotic Genome Annotation Pipeline (PGAP) [61]. Additionally, annotation with eggNOG [23], SignalP [24] and SecretomeP [25] was performed on the web (Supplementary Tables S1 and S2).

### 4.3. Co-Cultivations of LAB with K. pneumoniae

The MRS broth was inoculated with 1 mL of K. pneumoniae pre-culture and (1) 1 mL of *L. reuteri*LR1 pre-culture, (2) 1 mL of *L. rhamnosus* F pre-cultures and (3) 1 mL of *L. reuteri*LR1 and 1 mL of *L. rhamnosus* F pre-cultures. The final volume was adjusted with MRS broth to 20 mL, and incubations were performed under anaerobic conditions at 37 ± 1 °C for 24 and 48 h. All cultivations were performed in triplicate.

The viable cell count of K. pneumonia was performed on Nutrient agar (Mikrogen, Moscow, Russia), containing 18 g·L^−1^ of fish hydrolysate, 8 g·L^−1^ of NaCl, and 12 g·L^−1^ of agar, after cultivation under anaerobic conditions at 37 ± 1 °C for 24 h.

### 4.4. Co-Cultivations of LAB with Each Other

The MRS broth was inoculated with (1) 1 mL of *L. reuteri* LR1 pre-culture, (2) 1 mL of *L. rhamnosus* F pre-cultures and (3) 1 mL of *L. reuteri* LR1 and 1 mL of *L. rhamnosus* F pre-cultures. The final volume was adjusted with MRS broth to 20 mL, and incubations were performed under anaerobic conditions at 37 ± 1 °C for 24. All cultivations were performed in triplicate.

The viable cell count of *L. reuteri* LR1 was performed on MRS agar containing 0.002 g·L^−1^ of ampicillin after cultivation under anaerobic conditions at 37 ± 1 °C for 72 h. The viable cell count of *L. rhamnosus* F was performed, on nutrient agar containing 10 g·L^−1^ of tryptone, 5 g·L^−1^ of yeast extract, 1 g·L^−1^ of Tween 80, 2.6 g·L^−1^ of Na_2_HPO_4_, 5 g·L^−1^ of CH_3_COONa×3H_2_O, 2 g·L^−1^ of diammonium citrate, 0.2 g·L^−1^ of MgSO_4_, 0.05 g·L^−1^ of Mn_2_(SO_4_)_3_, and 13 g·L^−1^ of agar, after cultivation under anaerobic conditions at 37 ± 1 °C for 72 h.

### 4.5. Perpendicular Streak Test

At the first stage, the pre-culture of the first LAB (*L. reuteri* LR1 or *L. rhamnosus* F) was streaked on the MRS agar and incubated under anaerobic conditions at 37 °C for 24 h. At the second stage, the pre-culture of the second LAB (*L. rhamnosus* F or *L. reuteri*LR1, respectively) was streaked perpendicularly to the first LAB, and the plate was incubated under anaerobic conditions for another 24 h at 37 °C. The antagonistic interactions between LAB were assessed visually by the presence of a growth inhibition zone.

### 4.6. Exoproteomics Study

After 24 h of cultivation, bacterial cells were separated from the culture liquid by centrifugation at 8000× *g* for 40 min at 4 °C. The cultural liquids from the parallel cultivations were pulled together. All the following procedures were performed as described in [62]. In short, proteins were precipitated and separated by both mass and isoelectric point on two-dimensional (2D) gel. The obtained protein spots were further analyzed using Ultraflex II mass spectrometer (Bruker, Germany) with matrix-assisted laser desorption/ionization ion source and tandem time-of-flight mass analyzer (MALDI TOF/TOF MS/MS). The obtained data (both peptide fingerprints and sequences) were matched against an in-house database of proteins annotated in the genomes of *L. reuteri* LR1 and *L. rhamnosus* F (see the Section 4.2).

## 5. Conclusions

In conclusion, it was shown that as a part of their complex antagonistic system LAB can secrete proteins with potential antimicrobial activity against *K. pneumoniae* cells. The performed comparative MS analyses of the exoproteomes from co-cultivations of LAB with *K. pneumoniae* allowed the identification of several candidate antimicrobial proteins: NlpC/P60 endopeptidases, lytic transglycosylase, Zn-dependent protease (peptidase family M10), nucleoside hydrolase RihC, nucleotidase, ribonucleoside reductase and others. These enzymes are attractive targets for the discovery of novel antimicrobial compounds.

## Figures and Tables

**Table 1:** General data on the genome sequencing of *L. reuteri* LR1 and *L. rhamnosus* F.

*Limosilactobacillus reuteri* LR1 (GB Accession: GCA_018966925.1)				*Lacticaseibacillus rhamnosus* F (GB Accession: GCA_018966895.1)			
**Sequencing**				**Sequencing**			
Sequencing technology	Ion Torrent	Number of reads	3,358,179	Sequencing technology	Ion Torrent	Number of reads	3,817,756
		Mean read size, bp	209			Mean read size, bp	211
**Assembly**		**Structural annotation**		**Assembly**		**Structural annotation**	
Assembly size, bp	2,053,706	Genes (total):	2179	Assembly size, Mb	2,893,669	Genes (total):	2736
Overall coverage	100×	-Protein coding	1947	Overall coverage	100×	-Protein coding	2607
Number of contigs	319	-RNA coding	83	Number of contigs	57	-RNA coding	71
Longest contig, bp	64,232	-Pseudo genes	149	Longest contig, bp	310,149	-Pseudo genes	85
N50 contig size, bp	20,552	CRISPR arrays	0	N50 contig size, bp	144,365	CRISPR arrays	1
Mean contig size, bp	6437			Mean contig size, bp	44,023		

**Table 2:** Functional description of the proteins secreted by *L. reuteri* LR1 and *L. rhamnosus* F in the current study.

*Limosilactobacillus reuteri* LR1				
Protein ID	Functional Description/Possible Function	Functional Category	COG	Secretion
MBU5982788.1	L-lactate dehydrogenase (EC 1.1.1.27) that catalyzes the oxidation of (S)-lactate to the pyruvate.	Metabolism	C	None
MBU5983425.1	Possibly glyoxalase III (EC 4.4.1.5) that catalyzes conversion of methylglyoxal to lactate.	Metabolism	E	None
MBU5982312.1	Cysteine synthase A (EC 2.5.1.47) that catalyzes the formation of cysteine from O-acetylserine through the elimination of acetate and addition of hydrogen sulfide.	Metabolism	E	None
MBU5982880.1	Ornithine carbamoyltransferase (EC 2.1.3.3) that catalyzes the transfer of the carbamoyl group from carbamoyl phosphate to ornithine; produces L-citrulline	Metabolism	E	None
MBU5982566.1	Bacterial solute-binding protein (family 3) that participates in the active transport of amino acids across the cytoplasmic membrane via their delivery to the active-transport system.	Metabolism/Transport	E/T	Classical
MBU5982057.1	Ribonucleoside hydrolase RihC (EC 3.2.2.1) that catalyzes the hydrolysis of ribonucleosides with the formation of free ribose and the corresponding base.	Metabolism	F	None
MBU5982646.1	Nucleotidase (EC 3.1.3.5) that catalyzes the hydrolysis of ribonucleotides to the corresponding ribonucleosides.	Metabolism	F	Classical
MBU5983020.1	Ribonucleoside reductase (EC 1.17.4.1) that catalyzes the oxidation of ribonucleoside-diphosphates to the corresponding deoxyribonucleotides.	Metabolism	F	Non-classical
MBU5982282.1	Phosphoglycerate mutase (EC 5.4.2.11) that catalyzes conversion of bisphosphoglycerate to 3-phosphoglycerate at the eighth step of glycolysis.	Metabolism	G	None
MBU5982130.1	Citrate CoA-transferase (EC 2.8.3.10) that catalyzes the transfer of CoA from Acetyl-CoA to citrate. The enzyme is a component of EC 4.1.3.6 [citrate(pro-3-S)-lyase], which produces acetate and oxaloacetate from citrate.	Metabolism	H	None
MBU5982114.1	Chaperonin GroEL that prevents misfolding and promotes the refolding and proper assembly of unfolded polypeptides generated under stress conditions.	Protein folding	O	None
MBU5983126.1	Peptidase P60 (NlpC/P60; peptidase family C40; EC 3.4...) that catalyzes the hydrolysis of MurNAc-(L-alanine) bonds in peptidoglyucan and/or acts as γ-glutamyl DL-endopeptidase.	Cell wall degradation	M	Classical
MBU5982939.1	Metalloendopeptidase (peptidase family M23; EC 3.4...) that catalyzes the preferential hydrolysis of the glycine–glycine bonds in peptidoglycan.	Cell wall degradation	M	Classical
MBU5982718.1	Mannosyl-glycoprotein endo-beta-*N*-acetylglucosaminidase (GH73; EC 3.2.1.96) that catalyzes the hydrolysis of the bonds between GlcNAc residues and contiguous monosaccharides in peptidoglycan (contains carbohydrate-binding module family 50; CBM50).	Cell wall degradation	M	Classical
MBU5983247.1/MBU5983476.1	Putative adhesin; contains YSIRK-type signal peptide and LPXTG cell wall anchor domain; has multiple Rib/alpha-like repeats.	Cell wall adhesion	M	Classical
MBU5981906.1	Putative adhesin; contains LPXTG cell wall anchor domain.	Cell wall adhesion	M	Classical
***Lacticaseibacillus rhamnosus* F**				
**Protein ID**	**Functional Description/Possible Function**	**Functional Category**	**COG**	**Secretion**
MBU5979615.1	L-lactate dehydrogenase (EC 1.1.1.27) that catalyzes the oxidation of (S)-lactate to the pyruvate.	Metabolism	C	None
MBU5977590.1	D-lactate dehydrogenase (EC 1.1.1.28) that catalyzes the oxidation of (R)-lactate to the pyruvate.	Metabolism	C	None
MBU5977926.1	Malate dehydrogenase (EC 1.1.1.38) that catalyzes the oxidative decarboxylation of malate into pyruvate.	Metabolism	C	None
MBU5978913.1	Glucose-6-phosphate isomerase (EC 5.3.1.9) that catalyzes the isomerization of D-glucose 6-phosphate into D-fructose 6-phosphate at the second step of glycolysis.	Metabolism	G	None
MBU5977496.1	β-galactosidase (GH59; EC 3.2.1.23) that catalyzes the hydrolysis of β-galactosides into monosaccharides (contains FIVAR domain).	Metabolism	G	Classical
MBU5978705.1	Glyceraldehyde-3-phosphate dehydrogenase (type I; EC 1.2.1.12) that catalyzes interconversion of glyceraldehyde-3-phosphate and 1,3-diphosphoglycerate at the sixth step of glycolysis.	Metabolism	G	Non-classical
MBU5978932.1	Zn-dependent protease (peptidase family M10) that is homologous to the human matrix metallopeptidase capable of degrading extracellular matrix proteins.	Proteolysis	NA	Classical
MBU5978576.1	Cell envelope proteinase PrtR (peptidase family S8; EC 3.4.21.96) that degrades the extracellular proteins into oligopeptides.	Proteolysis	O	Classical
MBU5978925.1	Cold-shock protein that binds single-stranded nucleic acids and functions in a variety of processes that are related, for the most part, to post-translational gene regulation	Transcription	K	Non-classical
MBU5979459.1	Cell wall hydrolase P75 (NlpC/P60; peptidase family C40; EC 3.4...) that catalyzes the hydrolysis of MurNAc-(L-alanine) bonds in peptidoglyucan and/or acts as γ-glutamyl DL-endopeptidase.	Cell wall degradation	M	Classical
MBU5977346.1	Lytic transglycosylase (3D domain) that catalyzes cleavage of beta-1-4 bond between MurNAc and GlcNAc.	Cell wall degradation	M	Non-classical
MBU5978877.1	Viral capsid protein derived from the prophage integrated into the bacterial chromosome.	Unknown	NA	Non-classical

## Data Availability

The Whole Genome Shotgun projects were deposited at DDBJ/ENA/GenBank under the accessions JAHLXI000000000 and JAHLXH000000000 for *Limosilactobacillus reuteri* LR1 and *Lacticaseibacillus rhamnosus* F, respectively. The versions described in this paper are versions JAHLXI000000000.1 and JAHLXH000000000.1 for *L. reuteri* LR1 and *L. rhamnosus* F, respectively.

## Supplementary Materials

The following are available online at https://www.mdpi.com/article/10.3390/ijms222010999/s1.

## References

[1] Tanwar J., Das S., Fatima Z., Hameed S. (2014). Multidrug Resistance: An Emerging Crisis. Interdiscip. Perspect. Infect. Dis..

[2] Neubeiser A., Bonsignore M., Tafelski S., Alefelder C., Schwegmann K., Rüden H., Geffers C., Nachtigall I. (2020). Mortality attributable to hospital acquired infections with multidrug-resistant bacteria in a large group of German hospitals. J. Infect. Public Health.

[3] Burnham J.P., Olsen M.A., Kollef M.H. (2019). Re-estimating annual deaths due to multidrug-resistant organism infections. Infect. Control Hosp. Epidemiol..

[4] Nelson R.E., Hatfield K.M., Wolford H., Samore M.H., Scott R.D., Reddy S.C., Olubajo B., Paul P., Jernigan J.A., Baggs J. (2021). National Estimates of Healthcare Costs Associated With Multidrug-Resistant Bacterial Infections Among Hospitalized Patients in the United States. Clin. Infect. Dis..

[5] Silva D.R., de Sardi J.C.O., de Pitangui N.S., Roque S.M., da Silva A.C.B., Rosalen P.L. (2020). Probiotics as an alternative antimicrobial therapy: Current reality and future directions. J. Funct. Foods.

[6] Yang H., Sun Y., Cai R., Chen Y., Gu B. (2020). The impact of dietary fiber and probiotics in infectious diseases. Microb. Pathog..

[7] Vuotto C., Longo F., Donelli G. (2014). Probiotics to counteract biofilm-associated infections: Promising and conflicting data. Int. J. Oral Sci..

[8] Rakhmanova A., Khan Z.A., Shah K. (2018). A mini review fermentation and preservation: Role of Lactic Acid Bacteria. MOJ Food Process. Technol..

[9] Dimidi E., Cox S., Rossi M., Whelan K. (2019). Fermented Foods: Definitions and Characteristics, Impact on the Gut Microbiota and Effects on Gastrointestinal Health and Disease. Nutrients.

[10] Rozhkova I.V., Moiseenko K.V., Glazunova O.A., Begunova A.V., Fedorova T.V. (2020). 9 Russia and Commonwealth of Independent States (CIS) domestic fermented milk products. Food Science and Technology.

[11] De Vuyst L., Vandamme E.J. (1994). Antimicrobial Potential of Lactic Acid Bacteria. Bacteriocins of Lactic Acid Bacteria.

[12] Barzegari A., Kheyrolahzadeh K., Hosseiniyan Khatibi S.M., Sharifi S., Memar M.Y., Zununi Vahed S. (2020). The Battle of Probiotics and Their Derivatives Against Biofilms. Infect. Drug Resist..

[13] Vieco-Saiz N., Belguesmia Y., Raspoet R., Auclair E., Gancel F., Kempf I., Drider D. (2019). Benefits and Inputs From Lactic Acid Bacteria and Their Bacteriocins as Alternatives to Antibiotic Growth Promoters During Food-Animal Production. Front. Microbiol..

[14] Reis J.A., Paula A.T., Casarotti S.N., Penna A.L.B. (2012). Lactic Acid Bacteria Antimicrobial Compounds: Characteristics and Applications. Food Eng. Rev..

[15] Bengoechea J.A., Sa Pessoa J. (2019). Klebsiella pneumoniae infection biology: Living to counteract host defences. FEMS Microbiol. Rev..

[16] Wyres K.L., Holt K.E. (2018). Klebsiella pneumoniae as a key trafficker of drug resistance genes from environmental to clinically important bacteria. Curr. Opin. Microbiol..

[17] Navon-Venezia S., Kondratyeva K., Carattoli A. (2017). Klebsiella pneumoniae: A major worldwide source and shuttle for antibiotic resistance. FEMS Microbiol. Rev..

[18] González J.F., Hahn M.M., Gunn J.S. (2018). Chronic biofilm-based infections: Skewing of the immune response. Pathog. Dis..

[19] El-Mokhtar M.A., Hassanein K.M., Ahmed A.S., Gad G.F., Amin M.M., Hassanein O.F. (2020). Antagonistic Activities of Cell-Free Supernatants of Lactobacilli Against Extended-Spectrum β-Lactamase Producing Klebsiella pneumoniae and Pseudomonas aeruginosa. Infect. Drug Resist..

[20] Mogna L., Deidda F., Nicola S., Amoruso A., Del Piano M., Mogna G. (2016). In Vitro Inhibition of Klebsiella pneumoniae by Lactobacillus delbrueckii Subsp. delbrueckii LDD01 (DSM 22106). J. Clin. Gastroenterol..

[21] Raras T., Firman A., Kinanti I., Noorhamdani N. (2019). Anti-Biofilm Activity of Lactic Acid Bacteria Isolated from Kefir Against Multidrug-Resistant Klebsiella pneumoniae. J. Pure Appl. Microbiol..

[22] Lagrafeuille R., Miquel S., Balestrino D., Vareille-Delarbre M., Chain F., Langella P., Forestier C. (2018). Opposing effect of Lactobacillus on in vitro Klebsiella pneumoniae in biofilm and in an in vivo intestinal colonisation model. Benef. Microbes.

[23] Huerta-Cepas J., Szklarczyk D., Heller D., Hernández-Plaza A., Forslund S.K., Cook H., Mende D.R., Letunic I., Rattei T., Jensen L.J. (2019). eggNOG 5.0: A hierarchical, functionally and phylogenetically annotated orthology resource based on 5090 organisms and 2502 viruses. Nucleic Acids Res..

[24] Almagro Armenteros J.J., Tsirigos K.D., Sønderby C.K., Petersen T.N., Winther O., Brunak S., von Heijne G., Nielsen H. (2019). SignalP 5.0 improves signal peptide predictions using deep neural networks. Nat. Biotechnol..

[25] Bendtsen J.D., Kiemer L., Fausbøll A., Brunak S. (2005). Non-classical protein secretion in bacteria. BMC Microbiol..

[26] McFarland L.V. (2021). Efficacy of Single-Strain Probiotics Versus Multi-Strain Mixtures: Systematic Review of Strain and Disease Specificity. Dig. Dis. Sci..

[27] Kitts P.A., Church D.M., Thibaud-Nissen F., Choi J., Hem V., Sapojnikov V., Smith R.G., Tatusova T., Xiang C., Zherikov A. (2016). Assembly: A resource for assembled genomes at NCBI. Nucleic Acids Res..

[28] Mitrea L., Vodnar D.C. (2019). Klebsiella pneumoniae—A Useful Pathogenic Strain for Biotechnological Purposes: Diols Biosynthesis under Controlled and Uncontrolled pH Levels. Pathogens.

[29] Liu Y., Tang H., Lin Z., Xu P. (2015). Mechanisms of acid tolerance in bacteria and prospects in biotechnology and bioremediation. Biotechnol. Adv..

[30] Soltani S., Hammami R., Cotter P.D., Rebuffat S., Said L.B., Gaudreau H., Bédard F., Biron E., Drider D., Fliss I. (2021). Bacteriocins as a new generation of antimicrobials: Toxicity aspects and regulations. FEMS Microbiol. Rev..

[31] Prudêncio C.V., dos Santos M.T., Vanetti M.C.D. (2015). Strategies for the use of bacteriocins in Gram-negative bacteria: Relevance in food microbiology. J. Food Sci. Technol..

[32] Åvall-Jääskeläinen S., Palva A. (2005). Lactobacillus surface layers and their applications. FEMS Microbiol. Rev..

[33] Teame T., Wang A., Xie M., Zhang Z., Yang Y., Ding Q., Gao C., Olsen R.E., Ran C., Zhou Z. (2020). Paraprobiotics and Postbiotics of Probiotic Lactobacilli, Their Positive Effects on the Host and Action Mechanisms: A Review. Front. Nutr..

[34] Sánchez B., Urdaci M.C., Margolles A. (2010). Extracellular proteins secreted by probiotic bacteria as mediators of effects that promote mucosa–bacteria interactions. Microbiology.

[35] Kang Q., Zhang D. (2020). Principle and potential applications of the non-classical protein secretory pathway in bacteria. Appl. Microbiol. Biotechnol..

[36] Vermassen A., Leroy S., Talon R., Provot C., Popowska M., Desvaux M. (2019). Cell Wall Hydrolases in Bacteria: Insight on the Diversity of Cell Wall Amidases, Glycosidases and Peptidases Toward Peptidoglycan. Front. Microbiol..

[37] Kang M.-S., Lim H.-S., Oh J.-S., Lim Y., Wuertz-Kozak K., Harro J.M., Shirtliff M.E., Achermann Y. (2017). Antimicrobial activity of Lactobacillus salivarius and Lactobacillus fermentum against Staphylococcus aureus. Pathog. Dis..

[38] Xu Q., Sudek S., McMullan D., Miller M.D., Geierstanger B., Jones D.H., Krishna S.S., Spraggon G., Bursalay B., Abdubek P. (2009). Structural Basis of Murein Peptide Specificity of a γ-D-Glutamyl-L-Diamino Acid Endopeptidase. Structure.

[39] Kang S.J., Jun J.S., Moon J.A., Hong K.W. (2020). Surface display of p75, a Lactobacillus rhamnosus GG derived protein, on Bacillus subtilis spores and its antibacterial activity against Listeria monocytogenes. AMB Express.

[40] Singh N., Bhalla N. (2020). Moonlighting Proteins. Annu. Rev. Genet..

[41] Copley S.D. (2012). Moonlighting is mainstream: Paradigm adjustment required. BioEssays.

[42] Jeffery C.J. (1999). Moonlighting proteins. Trends Biochem. Sci..

[43] Jeffery C.J. (2018). Protein moonlighting: What is it, and why is it important?. Philos. Trans. R. Soc. B Biol. Sci..

[44] Kainulainen V., Korhonen T. (2014). Dancing to Another Tune—Adhesive Moonlighting Proteins in Bacteria. Biology.

[45] Jeffery C. (2018). Intracellular proteins moonlighting as bacterial adhesion factors. AIMS Microbiol..

[46] Jeffery J.C. (2019). Intracellular/surface moonlighting proteins that aid in the attachment of gut microbiota to the host. AIMS Microbiol..

[47] Joshi P., Gupta A., Gupta V. (2019). Insights into multifaceted activities of CysK for therapeutic interventions. 3 Biotech.

[48] Pal V.K., Bandyopadhyay P., Singh A. (2018). Hydrogen sulfide in physiology and pathogenesis of bacteria and viruses. IUBMB Life.

[49] Bernier S.P., Létoffé S., Delepierre M., Ghigo J.-M. (2011). Biogenic ammonia modifies antibiotic resistance at a distance in physically separated bacteria. Mol. Microbiol..

[50] Juarez G.E., Mateyca C., Galvan E.M. (2020). Proteus mirabilis outcompetes Klebsiella pneumoniae in artificial urine medium through secretion of ammonia and other volatile compounds. Heliyon.

[51] Wang X., Zheng Z., Dou P., Qin J., Wang X., Ma C., Tang H., Xu P. (2010). Cloning, expression, purification, and activity assay of proteins related to D-lactic acid formation in Lactobacillus rhamnosus. Appl. Microbiol. Biotechnol..

[52] Viana R., Yebra M.J., Galán J.L., Monedero V., Pérez-Martínez G. (2005). Pleiotropic effects of lactate dehydrogenase inactivation in Lactobacillus casei. Res. Microbiol..

[53] Valerio F. (2004). Production of phenyllactic acid by lactic acid bacteria: An approach to the selection of strains contributing to food quality and preservation. FEMS Microbiol. Lett..

[54] Hummel W., Schütte H., Kula M.R. (1985). d-2-hydroxyisocaproate dehydrogenase from Lactobacillus casei-A new enzyme suitable for stereospecific reduction of 2-ketocarboxylic acids. Appl. Microbiol. Biotechnol..

[55] Liu F., Tang C., Wang D., Sun Z., Du L., Wang D. (2021). The synergistic effects of phenyllactic acid and slightly acid electrolyzed water to effectively inactivate Klebsiella oxytoca planktonic and biofilm cells. Food Control.

[56] Cumby N., Davidson A.R., Maxwell K.L. (2012). The moron comes of age. Bacteriophage.

[57] Owen S.V., Canals R., Wenner N., Hammarlöf D.L., Kröger C., Hinton J.C.D. (2020). A window into lysogeny: Revealing temperate phage biology with transcriptomics. Microb. Genom..

[58] Biswas S., Keightley A., Biswas I. (2019). Characterization of a stress tolerance-defective mutant of Lactobacillus rhamnosus LRB. Mol. Oral Microbiol..

[59] Jarocki P., Komoń-Janczara E., Podleśny M., Kholiavskyi O., Pytka M., Kordowska-Wiater M. (2019). Genomic and Proteomic Characterization of Bacteriophage BH1 Spontaneously Released from Probiotic Lactobacillus rhamnosus Pen. Viruses.

[60] Fedorova T.V., Vasina D.V., Begunova A.V., Rozhkova I.V., Raskoshnaya T.A., Gabrielyan N.I. (2018). Antagonistic Activity of Lactic Acid Bacteria Lactobacillus spp. against Clinical Isolates of Klebsiella pneumoniae. Appl. Biochem. Microbiol..

[61] Tatusova T., DiCuccio M., Badretdin A., Chetvernin V., Nawrocki E.P., Zaslavsky L., Lomsadze A., Pruitt K.D., Borodovsky M., Ostell J. (2016). NCBI prokaryotic genome annotation pipeline. Nucleic Acids Res..

[62] Moiseenko K.V., Glazunova O.A., Savinova O.S., Vasina D.V., Zherebker A.Y., Kulikova N.A., Nikolaev E.N., Fedorova T.V. (2021). Relation between lignin molecular profile and fungal exo-proteome during kraft lignin modification by Trametes hirsuta LE-BIN 072. Bioresour. Technol..

